# Transcriptome-Wide Identification of miRNA Targets under Nitrogen Deficiency in *Populus* *tomentosa* Using Degradome Sequencing

**DOI:** 10.3390/ijms160613937

**Published:** 2015-06-18

**Authors:** Min Chen, Hai Bao, Qiuming Wu, Yanwei Wang

**Affiliations:** 1 National Engineering Laboratory for Tree Breeding, Beijing Forestry University, Beijing 100083, China; E-Mails: chenmin19920219@126.com (M.C.); holymagicmother@163.com (H.B.); qiumingwu930@126.com (Q.W.); 2 Key Laboratory of Genetics and Breeding in Forest Trees and Ornamental Plants, Ministry of Education, Beijing Forestry University, Beijing 100083, China; 3 College of Biological Sciences and Biotechnology, Beijing Forestry University, Beijing 100083, China

**Keywords:** degradome, miRNA, miRNA*, precursor, targets, *Populus tomentosa*

## Abstract

miRNAs are endogenous non-coding small RNAs with important regulatory roles in stress responses. Nitrogen (N) is an indispensable macronutrient required for plant growth and development. Previous studies have identified a variety of known and novel miRNAs responsive to low N stress in plants, including *Populus*. However, miRNAs involved in the cleavage of target genes and the corresponding regulatory networks in response to N stress in *Populus* remain largely unknown. Consequently, degradome sequencing was employed for global detection and validation of N-responsive miRNAs and their targets. A total of 60 unique miRNAs (39 conserved, 13 non-conserved, and eight novel) were experimentally identified to target 64 mRNA transcripts and 21 precursors. Among them, we further verified the cleavage of 11 N-responsive miRNAs identified previously and provided empirical evidence for the cleavage mode of these miRNAs on their target mRNAs. Furthermore, five miRNA stars (miRNA*s) were shown to have cleavage function. The specificity and diversity of cleavage sites on the targets and miRNA precursors in *P. tomentosa* were further detected. Identification and annotation of miRNA-mediated cleavage of target genes in *Populus* can increase our understanding of miRNA-mediated molecular mechanisms of woody plants adapted to low N environments.

## 1. Introduction

Nitrogen (N) is one of the most important nutrients required for plant growth and development. Not only does N constitute ~2% of plant dry matter as an essential component of key cellular molecules, such as proteins (amino acids), nucleic acids, chlorophyll, ATP, and secondary metabolites, it is also involved in pivotal regulatory biological processes, including carbon metabolism, amino acid metabolism, and protein synthesis [1]. In soil, N is taken up by plant roots in the form of nitrate, which is then reduced to ammonium by nitrate reductase (NR), and ammonium is incorporated into amino acids by the glutamate synthetase (GOGAT) cycle [2].

MicroRNAs (miRNAs) are endogenous small non-coding (~21 nucleotides [nts]) RNAs that play important roles in the regulation of gene expression at the transcriptional and post-transcriptional levels. Since the discovery of the miRNA s*let-7* and *lin-4* in *Caenorhabditis* *elegans*, many miRNAs have been detected in animals and plants [3,4]. In classical miRNA biogenesis in plants, the transcripts fold into hairpins to form primary miRNAs (pri-miRNAs). Through two sequential cleavages directed by Dicer-like 1 (DCL1), pri-miRNAs are first converted into precursor miRNAs (pre-miRNAs) and then into miRNA/miRNA star (miRNA*) duplexes, which are loaded onto the RNA-induced silencing complex (RISC). Finally, the miRNA strand serves as a guide to the RISC complex, which directs cleavage of the target mRNA at the paired region [5]. Recent studies have shown that miRNAs function as negative regulators of their target mRNAs through mRNA deadenylation and decay and translational repression [6]. In this way, miRNAs can regulate multiple processes of plant growth and development and can be involved in plant stress responses, such as drought, cold, mechanical, and heavy metal stresses and nutritional deficiency by altering the expression of transcription factors and protein coding genes [7]. For example, miRNAs and their corresponding target genes regulated by phosphate, sulfate, and nitrogen deficiency were globally identified in various plant species, such as *Arabidopsis*[8,9], rice [10,11], and maize [12,13]. It was reported that some genes coding nitrate transporters (AtNRT1.1 and AtNRT2.1) were involved in repressing lateral root initiation in response to high C:N ratios [9]. Moreover, previous studies demonstrated that miR169 was downregulatedin *Arabidopsis* under N deficiency, which caused an accumulation of its targets encoding nuclear factor-Y subunit A (NF-YA) family members [14]. In addition, in *Arabidopsis*, miR167/ARF8 and miR393/AFB3 regulatory patterns were shown to function in the N-responsive regulatory network, which regulates lateral root outgrowth [15].

Plant growth requires sufficient N, which is generally the most common limiting nutrient for crop growth and production. Limited N leads to significant changes in plant growth and development, such as root branching, leaf chlorosis, and decreased biomass [16]. As a result, crops typically require external fertilizer addition to fix sufficient N and ensure normal growth. However, woody plants such as *Populus* spp. generally absorb limited ammonium salts and nitrate from forest soil [17]. *Populus* *tomentosa* (the Chinese white poplar) is a fast-growing timber tree with excellent wood quality and resistance to various diseases and insects, and it plays a significant role in timber production for commercial use and ecological and environmental protection in northern China. In particular, *P. tomentosa* is emerging as a model organism for woody plants for a variety of growth processes and resistance studies in China [18].

However, there is little or no fertilization addition in the plantation of *P. tomentosa*, which is different from herbaceous plants with additional external fertilizer or symbiotic N fixation. As a consequence, a more comprehensive analysis of the nitrate-signaling pathway, which regulates the expression of a large set of genes responsible for morphological and physiological changes in *P. tomentosa* under conditions of N deficiency, is required. Previously, small RNA sequencing was used to investigate the differential expression of miRNAs in *Populus* under low N conditions [19], but experimentally validated targets of the miRNAs and the precise regulatory processes remain unknown. Recently, degradome analysis or parallel analysis of RNA ends (PARE), a high-throughput sequencing method, was developed to globally identify small RNA targets in *Arabidopsis* [20], *Oryza sativa* [21], *Populus* *trichocarpa* [22], and other plant species. Consequently, our investigation employed degradome sequencing to experimentally explore the target genes of various miRNAs responsive to nitrogen deficiency and increase our understanding of the mechanism of miRNA-mediated cleavage in woody plants.

## 2. Results

### 2.1. Construction of the Degradome Library and Sequencing Analysis in P. tomentosa

A degradome library was constructed from *P. tomentosa* plantlets grown under low N conditions. A total of 22,047,555 raw reads were generated using high-throughput degradome sequencing technology. After removing of low quality sequences, 21,906,451 reads (99.36%) remained (Table S1). The lengths of the filtered tags ranged from 18 to 24 nts, with 20 nts (59.61%) and 21 nts (38.37%) being the most abundant. A total of 5,114,992 unique reads representing 52.45% (9,751,810) of the filtered tags were mapped to the *P. tomentosa* genome. In addition, rRNA, cDNA sense, and unannotated sequences accounted for most of the reads, and miRNA-mRNA cleavage site prediction was thereafter analyzed based on unique reads that mapped to annotated genes in the reference genome (cDNA sense, 5,089,290, 52.19%) (Table S2).

### 2.2. Systematic Identification of miRNA Targets in P. tomentosa

In plants, the majority of miRNA-guided post-transcriptional regulation cleaves the target mRNA between the nts 10 and 11 in the complementary region of the miRNA:mRNA pair [5]. We used degradome sequencing to identify cleaved miRNA targets in *P. tomentosa* exposed to low N conditions*.* A total of 73,490 known transcripts and 356 miRNA sequences of *Populus* were used to annotate the degradome tags, and 85 (0.12%) target genes were verified in the N-deficiency degradome. Based on the relative abundances of target site reads compared with other sites in the gene model, the degradome peaks fell into five classes (categories 0, 1, 2, 3 and 4). We used target plots (*t*-plots) to present the abundances of the degradome signatures relative to their positions on the mRNA transcripts (Figure S1). Due to multiple cleavage sites on one gene, we identified a total of 91 category sites for these 85 unique targets including 64 protein-coding genes and 21 miRNA precursors (Table S3). In total, the majority of targets (62 out of 91) were grouped into categories of greater than the median confidence (categories 0, 1 and 2) in our degradome sequencing, accounting for 68%, similar with previous reports [23,24,25]. The relatively low confidence Cleaveland category, category 3 and 4 contain five and 24 targets, respectively, occupying a small proportion (32%, totally). Specifically, 64 protein-coding genes were targeted by 24 known miRNA families (15 conserved and nine non-conserved) and seven novel miRNAs in *P. tomentosa*. Of the identified 64 targets corresponding to 66 category sites, 13 (~20%) fell into category 0, while categories 1, 2, 3, and 4 had seven (11%), 29 (45%), four (6%), and 13 (20%) target sites, respectively, and category 2 was the most abundant (Table S3).

Global analysis of the targets for conserved, non-conserved, and novel miRNAs revealed that the number of target genes for each miRNA varied greatly, ranging from one to eight (Table S3). Among the miRNAs, pto-miR6439a was found to cleave eight mRNA transcripts, and 10 miRNAs (pto-miR395a, pto-miR395b-k, pto-miR396e-3p, pto-miR472b, pto-miR482a.2, pto-miR6427-3p, pto-miR6439b, and pto-miRS11) had four or more mRNA targets. Among all of the miRNAs detected based on our degradome sequencing, 13 conserved (33%), six non-conserved (46%), and six novel (75%) miRNAs had only one target gene. Unfortunately, a total of 35 target genes, targeted by 12 conserved miRNA families (16 unique miRNAs: miR156, miR160, miR166, miR167, miR169, miR172, miR396, miR472, miR476, miR478, miR482, and miR1446), five non-conserved miRNA families (miR6427, miR6439, miR6462, miR7828, and miR7841), and seven novel miRNAs (pto-miRS11, pto-SR22, pto-sR2, pto-smR5, pto-sM3, pto-M19, and pto-M53a) were shown to encode hypothetical proteins without precise biological function annotations, and require further experimental investigation (Table S3).

### 2.3. Annotation and Validation of Targets for Known Conserved miRNAs in P. tomentosa

Our degradome data identified 15 conserved miRNA families comprising 23 unique miRNAs targeting 33 protein-coding genes. This investigation identified seven specific target proteins for conserved miRNAs. Briefly, pto-miR159a/b targeted Potri.006G129900.1, encoding peroxidase 21 precursor; pto-miR169b-3p cleaved Potri.007G085200.1 (60S ribosomal protein L26B) and Potri.014G142600.1 (ORESARA 9 family protein); and pto-miR169ytargeted a gene encoding wound-responsive family protein (Potri.005G230600.1) (Table S3). Many studies have revealed the gene encoding transcription factor NF-YA as the universal target for miR169 across various plant species. In *Arabidopsis*, upregulation of the miR169 family promoted stress-induced early flowering by repressing the target gene encoding transcription factor AtNF-YA [26]. In maize, the miR169/NF-YA regulatory module is involved in response to various abiotic stresses, such as drought, salt, or abscisic acid (ABA) stress [27]. Reports have indicated that ORESARA 9 (ORE9) plays a vital role in signal transduction through interactions between its F-box motif and the plant Skp, Cullin and F-box containing (SCF) complex. Through the ubiquitin-proteasome pathway (proteolytic process), ORE9 limits leaf longevity by decreasing the target proteins required to delay leaf senescence in *Arabidopsis* [28]. In this N deficiency degradome analysis, pto-miR169y was shown to target genes encoding wound-responsive proteins, which are known to be induced in various biotic and abiotic stress responses, such as pathogen invasion in tobacco [29] and glutamate dehydrogenase (GDH) deficiency in *Arabidopsis* [30].

The low affinity sulfate transporter (LAST) 3 family protein encoded by 13 genes in a gene cluster was identified as the target of two distinct miRNAs (pto-miR395a and pto-miR395b-k). miRNAs pto-miR396g-5p and pto-miR477a-3p were shown to target Potri.017G062800.2 (shikimate kinase [SK] family protein) and Potri.001G289500.1 (tubulin alpha chain family protein), respectively(Table S3). It has been shown that SK family protein catalyzes the phosphorylation of shikimate to generate shikimate 3-phosphate, a significant step of the shikimate pathway, which is the major route of *de novo* synthesis of aromatic compounds and secondary metabolites in plants [31]. In addition, the tubulin alpha chain family protein typically associates with β-tubulin subunits, forming a tubulin heterodimer. The microtubules arranged by tubulin heterodimers play important roles in cell wall assembly, tissue patterning, organ shape, and direction of organ growth [32]. In this study, miRNA pto-miR482a.2 was shownto target six genes, all encoding hypothetical proteins that had not yet been fully characterized. However, eugene3.00102261, encoding disease resistance proteins, was validated to be the target of ptc-miR482.2, suggesting that ptc-miR482.2 may affect resistance to biotic and abiotic stresses in plants by regulating the expression of disease resistance proteins [33]. Unfortunately, 20 target genes for 17 conserved miRNAs were found to encode uncharacterized proteins (Table S3).

In this investigation, there was a common trend in the distribution of the target categories: the targets of the same miRNA family typically belonged to the same category. For pto-miR475, all targets among different family members fell into category 4, and for pto-miR395, most targets (11) belonged to category 0, with the exception of Potri.002G092400.3 (category 2) (Table S3).

### 2.4. Annotation and Validation of Targets of Known Non-Conserved miRNAs in P. tomentosa

By comparing our data with miRBase 20.0 (http://microran.sanger.ac.uk/sequence/index.html/), 10 unique miRNAs (pto-miR1449, pto-miR6427-3p, pto-miR6439a, pto-miR6439b, pto-miR6462c-5p, pto-miR6463, pto-miR6470, pto-miR6479, pto-miR7828, and pto-miR7841) were defined as known non-conserved plant miRNAs that have been previously identified only in *P. trichocarpa*. These 10 miRNAs were shown to target 24 mRNA transcripts. Category 2 comprised 71% (17 out of 24) of the non-conserved miRNAs targets, followed by category 4, which accounted for ~17% (four out of 24). Two target genes were classified as category 0 and one as category1 (Table S3). Notably, some non-conserved miRNAs tended to cleave multiple genes belonging to one gene family. For example, pto-miR6439a targeted eight transcripts at the same cleavage site, all falling into category 2; moreover, the identified targets for pto-miR6427-3p and pto-miR6439b also exhibited this characteristic.

Although there were six (25%) hypothetical proteins, seven characterized target proteins of non-conserved miRNAs were identified using degradome analysis. The helicase domain-containing family protein (Potri.005G050500.1, targeted by pto-miR1449) is universal in various plant species, in which it is indispensable in replication or transcription of DNA or RNA. There are two target genes for pto-miR6427-3p, Potri.001G014000.2 and Potri.001G014000.3, both encoding lipoyltransferase 2. pto-miR6439a and pto-miR6439b were found to cleave seven target genes coding germin-like protein 1 (GLP1) and four target genes encoding 26S protease regulatory subunit 7, respectively. A previous investigation showed that GLP1 may play a significant role in the leaf intercellular space and leaf architectural changes in tobacco [34]. However, transgenic *Arabidopsis* with the *BvGLP-1* gene was enhanced in the defense against the invasion of *Verticillium* *longisporum* and *Rhizoctonia* *solani* [35], suggesting that GLP1 might be involved in plant defense responses (Table S3).

Targets encoding a C2 domain-containing protein (Potri.009G134200.4) and a glycine-rich RNA-binding protein (GRP) (Potri.010G228700.1) were spliced by pto-miR6463 and pto-miR6470, respectively. As an important component of an intact protein, the C2 domain has an indispensable function in biological processes. For example, the C2 domain can aid in phospholipid binding and targeting proteins to cell membranes through lipid selectivity [36], and a calcium-dependent phospholipid-binding C2 domain-containing protein includes BAP1 and BAP2, which function as inhibitors of programmed cell death (PCD) in *Arabidopsis* [37]. GRPs may have important effects on plant development and biotic and abiotic stress resistance (such as low/high temperature, high salt, heavy metals, flooding, or drought) in *Arabidopsis* [38], rice [39], maize [40], and other plant species. In addition, pto-miR6479 was found to target genes encoding an oxidoreductase family protein (Potri.001G452600), which is an enzyme catalyzing the transfer of electrons from reductants (electron donors) to oxidants (electron acceptors) (Table S3).

### 2.5. Annotation and Validation of the Targets of Novel miRNAs in P. tomentosa

In this study, seven novel miRNAs (pto-miRS11, pto-SR22, pto-sR2, pto-smR5, pto-sM3, pto-M19, and pto-M53a) found in *P. tomentosa* in our previous investigation were shown to cleave eight targeted gene transcripts [41,42]. Among them, six targets were distributed among four categories, and one protein encoding gene belonged to categories 1 (17%), 2, and 3, respectively, whereas three cleaved targets fell into category 4 (50%). Especially, the Potri.010G066400.3 could be targeted by pto-miRS11 at seven sites, falling into category 2 (2, 29%), 3 (2, 29%), and 4 (3, 42%) (Table S3). Unfortunately, all target genes encoded uncharacterized proteins, and further experimental studies are required to determine their biological functions.

### 2.6. Detection and Validation of Targets of N-Responsive mRNAs in P. tomentosa

A previous investigation determined that the expression of 34 unique miRNAs (30 downregulated and four upregulated miRNAs) was significantly altered in *P. tomentosa* under low N stress [20]. Among them, the targets of 11 responsive miRNAs were identified in our N deficiency degradome, which provided further empirical evidence of bona fide targets cleaved by miRNAs. Specifically, eight unique miRNAs including seven conserved miRNAs (pto-miR159a/b, pto-miR160e-3p, pto-miR166a-m, pto-miR169a/b-5p/c, pto-miR172a/b-3p/c/f, pto-miR396c/d/e-5p, and pto-miR475a-3p/b-3p) and one non-conserved miRNA (pto-miR6427-3p) were downregulated, whereas three miRNAs (pto-miR396a/b, pto-miR396e-3p, and pto-miR395b-k) were upregulated under N deficiency stress [20] (Table S4). A total of 26 targets comprising 16 protein coding genes and 10 precursors were determined to be cleaved by these 11 N-responsive miRNAs. Among them, pto-miR159a/b targeted Potri.006G129900.1 encoding a peroxidase 21 precursor family protein, and pto-miR6427-3p could target four genes encoding LIPOYLTRANSFERASE 2 family proteins, whereas low affinity sulfate transporter 3 encoded by seven genes of a cluster was targeted by pto-miR395b-k. Furthermore, several target genes of these N-responsive miRNAs were identified to be hypothetical proteins that need further investigation. In addition, five miRNAs (pto-miR172a/b-3p/c/f, pto-miR396a/b, pto-miR396c/d/e-5p, pto-miR396e-3p, and pto-miR475a-3p/b-3p) were found to target 10 miRNA precursors (Table S4).

### 2.7. Specificity and Diversity of Cleavage Sites

**Figure 1:**
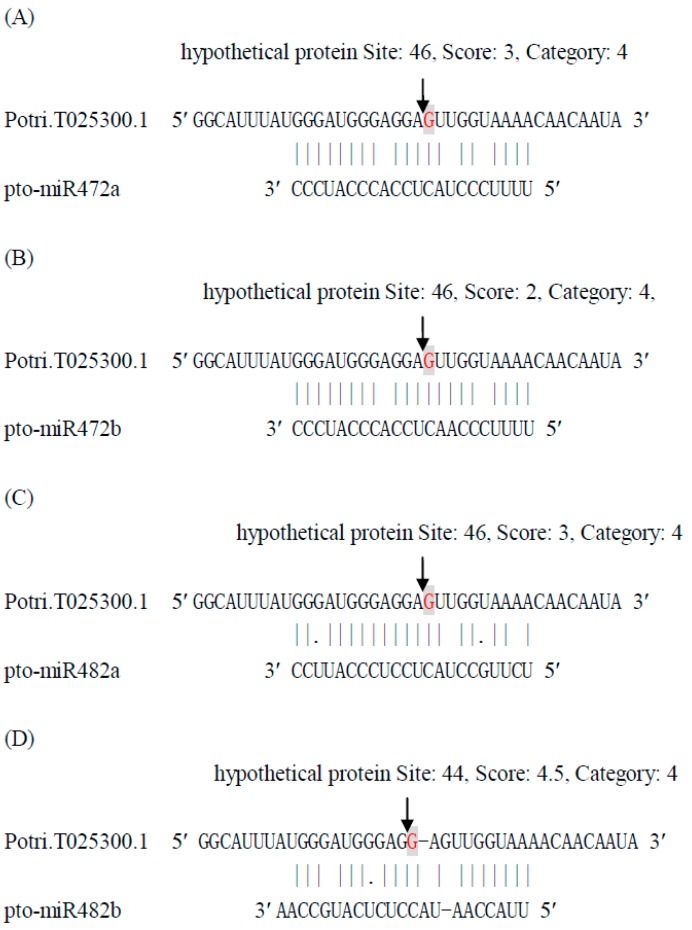
The cleavage of Potri.T025300.1 by pto-miR472a (**A**); pto-miR472b (**B**); pto-miR482a.2 (**C**); and pto-miR482b-3p (**D**). The solid lines and dots in the miRNA:mRNA alignments indicate matched RNA base pairs and GU mismatches, respectively. The arrows show the cleavage sites and the red shaded letters indicate the cleaved bases identified by degradome sequencing.

Based on sequence homology, different members of the same miRNA family can target the same transcripts at the same cleavage site. However, our degradome sequencing indicated that a target gene could be cleaved by distinct miRNAs at different positions within the mRNA transcript. For example, the transcript Potri.T025300.1 could be cleaved by pto-miR472a, pto-miR472b, and pto-miR482a.2 at position 46, and by pto-miR482b-3p at position 44 (Figure 1). Similar cleavage patterns were identified for Precursor-MIR398c, Precursor-MIR475b, Precursor-sR3, and Precursor-sR6a (Table S5).

**Figure 2:**
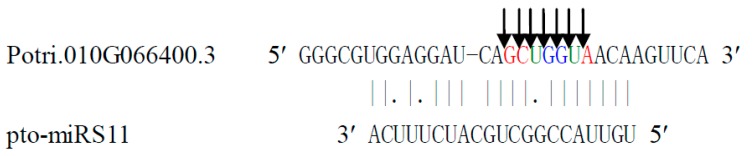
The cleavage of Potri.010G066400.3 targeted by pto-miRS11 at seven cleavage sites, falling into three categories. The solid lines and dots in the miRNA:mRNA alignments indicate matched RNA base pairs and GU mismatches, respectively. The arrows indicate the cleavage sites and colored letters show the cleaved based identified by degradome sequencing. The three red letters (sites 66 [G], 67 [C], and 72 [A]) indicate category 4 cleavage with count 1, the two green letters (sites 68 [U] and 71 [U]) category 2 cleavage with count 3, and the two blue letters (sites 69 [G] and 70 [G]) category 3 cleavage with count 2.

Generally, miRNAs cleave target mRNAs at specific and fixed sites, but occasionally cleavage at different sites within the transcript also occurs. For example, pto-miRS11 was found to cleave the transcript (010G066400.3) at different positions, resulting in different cleaved target categories. Specifically, pto-miRS11 cleaved 010G066400.3 at positions 68 and 71, falling into category 2, at positions 69 and 70, falling into category 3, and at positions 66, 67, and 72, falling into in category 4 (Figure 2 and Table S5).

### 2.8. Identification of miRNA Precursor in P. tomentosa Using Degradome Sequencing

**Figure 3:**
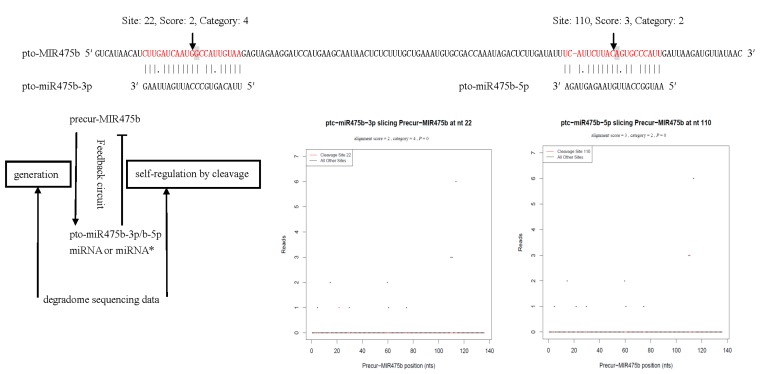
The cleavage of precursor-MIR475b targeted by pto-miR475b-3p and pto-miR475b-5p and the feedback regulatory circuit between Precur-MIR475b and pto-miR475b-3p or pto-miR475b-5p. Notes: Clearly, pre-MIR475b can be cleaved by DCL1, generating pto-miR475b-3p and pto-miR475b-5p. Our degradome sequencing revealed that pto-miR475b-3p and pto-miR475b-5p direct cleavage between nucleotides 10 and 11 of the complementary region of the miRNA:mRNA pair, splicing their target Precur-MIR475b to negatively regulate its abundance. Thus, there might be a regulatory feedback loop between Precur-MIR475b and pto-miR475b-3p or pto-miR475b-5p (miRNA or miRNA*), which could function as a buffering system to maintain mature miRNAs or miRNA*s at an appropriate level. The sequences in red letters show the miRNA and miRNA* of pto-miR475b, respectively. The solid lines and dots in the miRNA:mRNA alignments indicate matched RNA base pairs and GU mismatches, respectively. The arrows indicate the cleavage sites and the shaded letters indicate the cleaved bases identified by degradome sequencing.

In addition to specific miRNA targets, degradome sequencing can be used to detect miRNA precursor processing and the generation of mature miRNAs or miRNA*s [43]. In the degradome library, a total of 21 precursors were identified for 10 known miRNA families and one novel miRNA. Seven conserved miRNA families (miR168, miR172, miR319, miR396, miR398, miR408, and miR475) containing 18 miRNAs cleaved 16 precursors, while three non-conserved miRNAs (miR1447, miR1450, and miR6421) and one novel miRNA (pto-miRS12) targeted three and two precursors, respectively (Table S3). Furthermore, the feedback regulatory circuit between precursors and their miRNAs or miRNA*s has been previously identified in *Arabidopsis* [20] and rice [44]. Interestingly, pto-MIR475b was found to be cleaved by pto-miR475a-3p/b-3p at nt 22 but by pto-miR475a-5p/b-5p at nt 110, indicative of the feedback regulatory role of miRNAs/miRNA*s on their precursors (Figure 3).

It is known that precursors can be cleaved by DCL1 to produce miRNAs or miRNA*s. Therefore, based on degradome sequencing, sequences identical to miRNAs or miRNA*s and their corresponding cleavage sites may be identified within their precursors. Our investigation identified a total of 45 precursor miRNAs corresponding tomiRNAs or miRNA*s (Table 1), further elucidating precursor processing and providing more empirical evidence that specific miRNAs or miRNA*sare indeed generated from the cleaved precursors. For example, the precursor of pto-MIR394awasfound to become sliced and generate pto-miR394a-5p (TTGGCATTCTGTCCACCTCC) at positions 38–57 and pto-miR394a-3p (CTGTTGGTCTCTCTTTGTAA) at positions 117–136. Based on our local blast analysis, the precursor of pto-MIR6421 generated pto-miR6421-5p (TCCCTTACAATCTACTCTTTC) at positions 18–38 (Table 1). Based on analysis of the precursor processing, our investigation provides further empirical evidence that miRNAs and miRNA*s are generated from their precursors through DCL1-mediated cleavages; furthermore, miRNAs or miRNA*s can negatively regulate their pre-miRNAs by cleavage at 10th or 11th within the complementary region of the miRNA (miRNA*):miRNA pairs.

**Table 1:** Identification of miRNAs generated from precursor miRNAs by local blast analysis.

Pre-miRNA	MiRNA Sequence	Count	Letters	Cleavage Sites
pto-MIR156a	TGACAGAAGAGAGTGAGCAC	10	20	11–30
pto-MIR156b	TGACAGAAGAGAGTGAGCAC	10	20	11–30
pto-MIR156c	TGACAGAAGAGAGTGAGCAC	10	20	11–30
pto-MIR156d	TGACAGAAGAGAGTGAGCAC	10	20	11–30
pto-MIR156e	TGACAGAAGAGAGTGAGCAC	10	20	11–30
pto-MIR156f	TGACAGAAGAGAGTGAGCAC	10	20	11–30
pto-MIR156g	TTGACAGAAGATAGAGAGCAC	84	21	11–31
pto-MIR156h	TTGACAGAAGATAGAGAGCAC	84	21	11–31
pto-MIR156i	TTGACAGAAGATAGAGAGCAC	84	21	11–31
pto-MIR156j	TTGACAGAAGATAGAGAGCAC	84	21	11–31
pto-MIR156k	TGACAGAAGAGAGGGAGCA	2	20	11–29
pto-MIR159a	TTTGGATTGAAGGGAGCTCTA	408	21	154–174
pto-MIR159d	CTTGGATTGAAGGGAGCTCCT	35	21	165–185
pto-MIR162a	TCGATAAACCTCTGCATCCAG	34	21	78–98
pto-MIR167e	TGAAGCTGCCAGCATGATCTG	2	21	11–31
pto-MIR167h	TGAAGCTGCCAACATGATCTG	2	21	11–31
pto-MIR168a	TCGCTTGGTGCAGGTCGGGAA	2	21	11–31
pto-MIR168b	TCGCTTGGTGCAGGTCGGGAA	2	21	11–31
pto-MIR169o	AAGCCAAGGATGACTTGCCTG	10	21	11–31
pto-MIR171e	TGATTGAGCCGTGCCAATATC	1	21	65–85
pto-MIR171f	TGATTGAGCCGTGCCAATATC	1	21	101–121
pto-MIR171g	TGATTGAGCCGTGCCAATATC	1	21	71–91
pto-MIR171h	TGATTGAGCCGTGCCAATATC	1	21	71–91
pto-MIR171i	TGATTGAGCCGTGCCAATATC	1	21	106–126
pto-MIR172d	GGAATCTTGATGATGCTGCAT	2	21	153–173
pto-MIR172e	GGAATCTTGATGATGCTGCAT	2	21	104–124
pto-MIR319a	TTGGACTGAAGGGAGCTCCC	11	20	160–179
pto-MIR319b	TTGGACTGAAGGGAGCTCCC	11	20	157–176
pto-MIR319c	TTGGACTGAAGGGAGCTCCC	11	20	168–187
pto-MIR319d	TTGGACTGAAGGGAGCTCCC	11	20	165–184
pto-MIR394a	TTGGCATTCTGTCCACCTCC	2	20	38–57
pto-MIR394a	CTGTTGGTCTCTCTTTGTAA	1	20	117–136
pto-MIR394b	TTGGCATTCTGTCCACCTCC	2	20	38–57
pto-MIR394b	CTGTTGGTCTCTCTTTGTAA	1	20	117–136
pto-MIR396a	TTCCACAGCTTTCTTGAACTG	33	21	11–31
pto-MIR396b	TTCCACAGCTTTCTTGAACTG	33	21	11–31
pto-MIR396c	TTCCACAGCTTTCTTGAACTT	9	21	11–31
pto-MIR396d	TTCCACAGCTTTCTTGAACTT	9	21	20–40
pto-MIR396e	TTCCACAGCTTTCTTGAACTT	9	21	11–31
pto-MIR397a	TCATTGAGTGCAGCGTTGATG	6	21	11–31
pto-MIR408	ATGCACTGCCTCTTCCCTGGC	149	21	76–96
pto-MIR475d	TTACAGAGTCCATTGATTAAG	2	21	77–97
pto-MIR482a	TCTTGCCTACTCCTCCCATT	3	20	68–87
pto-MIR1444a	TCCACATTCGGTCAATGTTC	2	20	59–78
pto-MIR6421	TCCCTTACAATCTACTCTTTC	1	21	18–38
pto-MIR6457b	TTAGTTTGGCAGCCTCTTCTC	8	21	151–171
pto-MIR6460	TGATATGTGGCATTCAATCGA	1	21	93–113

### 2.9. Identification of Biologically Functional miRNA* Strands in P. tomentosa by Degradome Analysis

**Figure 4:**
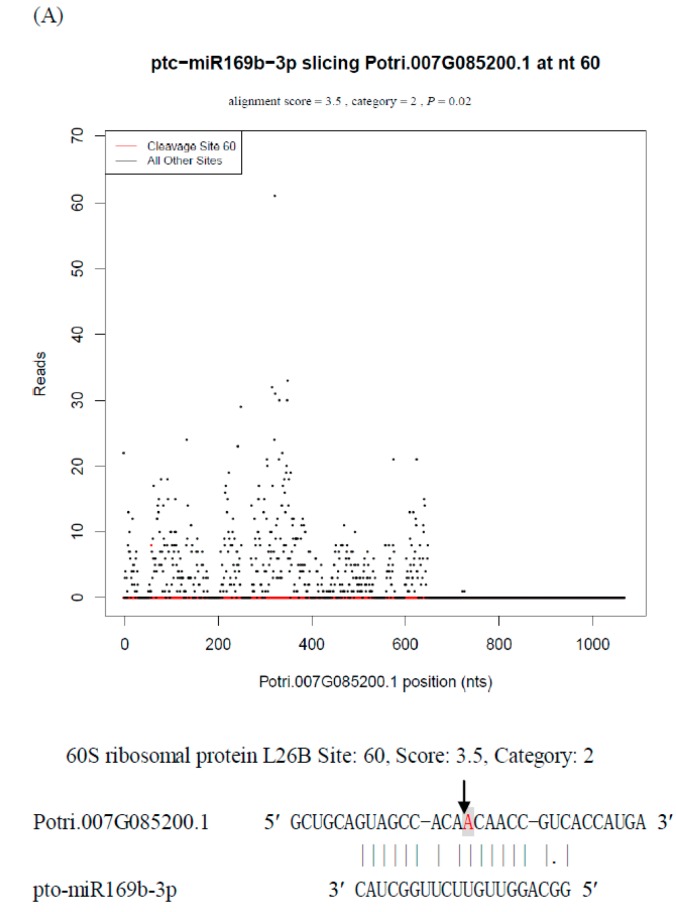

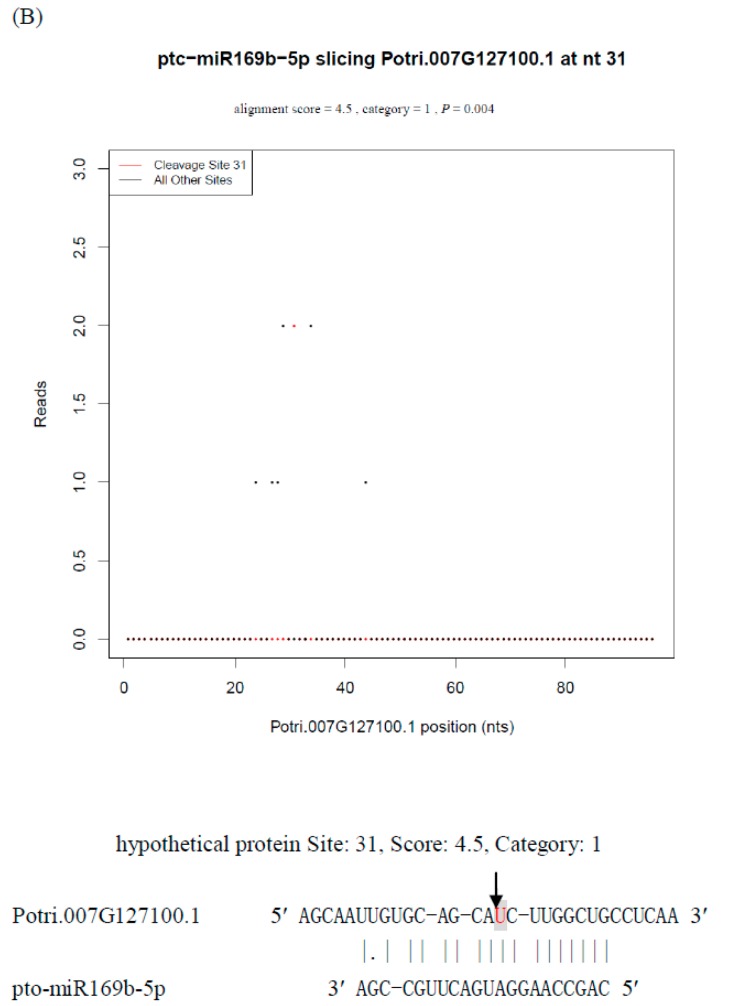
Target plots (*t*-plots) and cleavage site of Potri.007G085200.1 targeted by pto-miR169b-3p (**A**) and Potri.007G127100.1 targeted by pto-miR169b-5p (**B**). Red dots indicate signatures consistent with miRNA-directed cleavage. The solid lines and dots in the miRNA:mRNA alignments indicate matched RNA base pairs and GU mismatches, respectively. Below the *t*-plots, the cleavage sites are shown by the arrows and the cleaved bases are indicated by shaded letters.

Interestingly, our degradome sequencing revealed five unique miRNAs (pto-miR169b-3p/b-5p, pto-miR396e-3p/e-5p, pto-miR398c-3p/c-5p, pto-miR475a-3p/a-5p, and pto-miR475b-3p/b-5p), in which both of their duplex strands possessed cleavage function (Table 2). Moreover, our degradome sequencing identified potential targets of both strands of one miRNA duplex. For example, pto-miR169b-3p was identified to target three genes that encode the 60S ribosomal protein L26B (Potri.007G085200.1), a hypothetical protein (Potri.010G155300.1), and an ORE9 family protein (Potri.014G142600.1), respectively, while pto-miR169a/b-5p/c cleaved genes encoding uncharacterized proteins (Potri.007G127100.1) (Figure 4). These degradome data further revealed the specific biological functions of miRNA* strands in specific protein physiological processes.

**Table 2:** Identification of functional miRNAs and miRNA* strands.

MiRNA	MiRNA or miRNA* Sequence	Target Gene	C-Sites	Category	Raw Tags	Score
pto-miR169b-5p^1^	CAGCCAAGGATGACTTGCCGA	Potri.007G127100.1	31	1	2	4.5
pto-miR169b-3p	GGCAGGTTGTTCTTGGCTAC	Potri.007G085200.1	60	2	8	3.5
pto-miR169b-3p	GGCAGGTTGTTCTTGGCTAC	Potri.010G155300.1	45	2	2	4.5
pto-miR169b-3p	GGCAGGTTGTTCTTGGCTAC	Potri.014G142600.1	38	2	2	4
pto-miR396e-5p^1^	TTCCACAGCTTTCTTGAACTT	Precur-sR6a	127	2	7	3.5
pto-miR396e-3p^2^	CTCAAGAAAGCTGTGGGAGA	Precur-MIR396a	19	2	5	1.5
pto-miR396e-3p^2^	CTCAAGAAAGCTGTGGGAGA	Precur-MIR396b	19	2	5	1.5
pto-miR396e-3p^2^	CTCAAGAAAGCTGTGGGAGA	Precur-MIR396e	19	4	1	2
pto-miR396e-3p^2^	CTCAAGAAAGCTGTGGGAGA	Precur-sR3	22	2	5	1.5
pto-miR396e-3p^2^	CTCAAGAAAGCTGTGGGAGA	Precur-sR6a	19	4	1	2
pto-miR398c-5p	GGAGCGACCTGAAATCACATG	Precur-MIR398b	65	1	2	3
pto-miR398c-5p	GGAGCGACCTGAAATCACATG	Precur-MIR398c	77	2	2	3
pto-miR398c-3p	TGTGTTCTCAGGTCGCCCCTG	Precur-MIR398c	22	0	49	3
pto-miR475a-3p/b-3p^1^	TTACAGTGCCCATTGATTAAG	Precur-MIR475a	15	4	1	2
pto-miR475a-3p/b-3p^1^	TTACAGTGCCCATTGATTAAG	Precur-MIR475b	22	4	1	2
pto-miR475a-3p/b-3p^1^	TTACAGTGCCCATTGATTAAG	Precur-MIR475d	19	4	1	3
pto-miR475a-5p/b-5p	AATGGCCATTGTAAGAGTAGA	Precur-MIR475b	110	2	3	3

The superscript “1” indicates the down-regulated miRNAs in response to N starvation, whereas “2” shows the up-regulated miRNAs in response to N starvation.

## 3. Discussion

### 3.1. N-Responsive miRNA-Mediated Targets

When partially or completely complementary to target mRNAs, miRNAs can regulate gene expression through miRNA-mediated translational repression or target degradation. Using degradome sequencing, the mRNA targets and patterns of mRNA degradation had been successfully detected and validated in rice [44] and *Arabidopsis* [45]. According to our previous study on *P. tomentosa* by Ren *et al.* (2015), seven conserved unique miRNAs (pto-miR159a/b, pto-miR160e-3p, pto-miR166a-m, pto-miR169a/b-5p/c, pto-miR172a/b-3p/c/f, pto-miR396c/d/e-5p, and pto-miR475a-3p/b-3p) and one non-conserved miRNA (pto-miR6427-3p) were downregulated under low N stress. In addition, three conserved miRNAs (pto-miR396a/b, pto-miR396e-3p, and pto-miR395b-k) were upregulated under low N stress [20], and the corresponding targets were identified in our degradome sequencing (Table S4).

**Figure 5:**
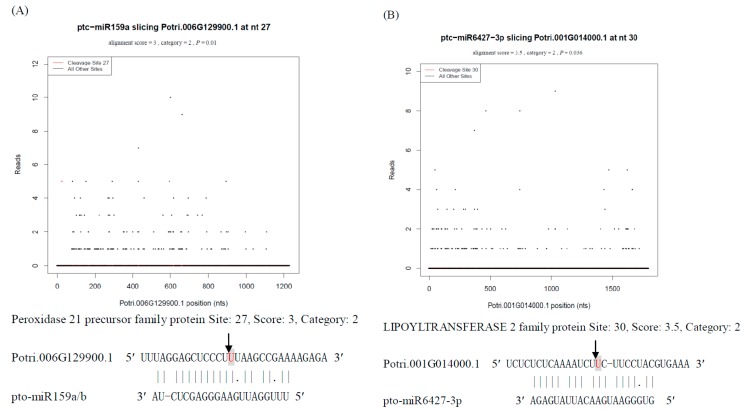

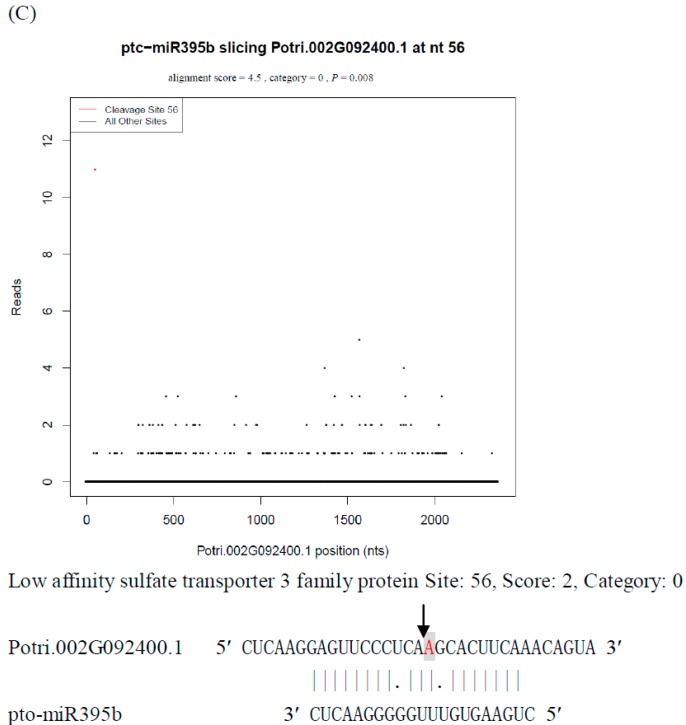
Target plots (*t*-plots) for three N-responsive miRNA targets confirmed by degradome sequencing (**A**) ptm-miR159a; (**B**) pto-miR395b; (**C**) pto-miR6427-3p. Red dots indicate signatures consistent with miRNA-directed cleavage. The solid lines and dots in the miRNAmRNA alignments indicate matched RNA base pairs and GU mismatches, respectively. Below the *t*-plots, the cleavage sites are shown by the arrows and the cleaved bases are indicated by shaded letters.

The gene encoding the R2R3 MYB domain transcription factor is a conserved target of the miR159 family in plants. In *Arabidopsis*, miR159a/b have been verified to target encoding R2R3 MYB to repress growth and advance programmed cell death through the GA (Gibberellic acid) signal transduction pathway [46]. A decrease in stu-miR159s resulted in an increase in *GAMyb*-like genes, thus improving drought tolerance in the potato [47]. Our degradome sequencing showed that pto-miR159a/b cleaved target gene encoding peroxidase (Figure 5a), an enzyme that catalyzes reduction reactions conferring resistance to oxidative stress in biological processes involving hydrogen peroxide or other organic hydroperoxides as electron donors.

In rice, lipoyl-protein ligase A (OsLPLA) catalyzes the covalent bonding of lipoic acid to lipoate-dependent enzymes, such as pyruvate dehydrogenase (PDH), and alpha-ketoglutarate dehydrogenase (KGDH) [48]. Similarly, in *Arabidopsis*, the mitochondrial-located LIP2 can catalyze an identical reaction to form key enzyme complexes that function in various central metabolic processes [49]. In *P. tomentosa*, the downregulated miRNAs pto-miR6427-3p targeted genes encoding a lipoyltransferase 2 family protein (Table S3; Figure 5b), whose function requires further investigation.

Generally, the uptake and internal concentration of one nutrient may be altered by the relative abundance of other nutrients, which may be related to an altered metal homeostasis response [50,51]. Previous studies have revealed that N and S metabolic pathways interact with each other, and that miR395 is responsive to a variety of environmental stresses, such as salt, drought, oxidative stress, and inorganic phosphate (*P*_i_) starvation [52]. In our investigation, miR395 was found to cleave target genes encoding the LAST protein (Table S3; Figure 5C). According to a previous report by Ren *et al.*, pto-miR395b-k was upregulated in *P. tomentosa* in response to low N stress, possibly causing a decrease in the LAST3 family protein, which indicates that a deficiency in N may suppress the uptake of sulfate. Studies in *Arabidopsis* and on cadmium stress in *Brassica napus* revealed that constitutive LAST expression may be involved in sulfate uptake from soil and roots to shoots transport [53,54].

Interestingly, certain miRNAs are involved in diverse pentatricopeptide repeat (PPR) functions in response to various biotic and abiotic stresses. For example, the well-known cold-responsive miRNAs (miR160, miR169, and miR396) in *Arabidopsis* are induced by viral infection [55]. These three degradome-validated miRNAs (pto-miR160e-3p, pto-miR169a/b-5p/c, pto-miR396c/d/e-5p) were all found to be depressed under N deficiency in the previous study [20]. In addition, ptc-miR475a and b were downregulated in response to cold stress [33], whereas pto-miR475a-3p and b-3p were reduced in response to low N stress.

### 3.2. Analysis of miRNA Precursor Processing in P. tomentosa Using Degradome Sequencing

In the process of miRNA biogenesis, DCL1 converts a pri-miRNA to a pre-miRNA and then to a miRNA/miRNA* duplex via two sequential cleavages. The synthesized miRNA/miRNA* duplex is subsequently guided to the argonaute (AGO)-associated RISC, and the miRNA* is degraded [6]. Using the degradome data from rice, Li *et al.* (2010) investigated DCL1-mediated miRNA precursor processing in detail. The detection of cleavage signals in the middle of the miRNA/miRNA* loci on pre-miRNAs indicated that miRNA/miRNA*-mediated self-regulation of their host precursors may occur in *Arabidopsis* [20] and rice [44]. Therefore, in addition to the classical DCL1-mediated cleavages and HST-mediated exportation from the nucleus to regulate the expression of miRNA genes at the post-transcriptional level, there are negative feedback regulatory loops between some precursors and their miRNAs or miRNA*s, which could function as a buffering system to maintain the miRNAs or miRNA*s at appropriate levels. Specifically, over-produced precursors generate more miRNAs or miRNA*s, and these excessive miRNAs or miRNA*s can direct mid-sequence cleavages of miRNAs/miRNA* precursors through complementary pairing, limiting the biogenesis of further miRNAs or miRNA*s, thus ensuring the appropriate abundance of functional miRNAs or miRNA*s [43]. In this investigation, pto-MIR475b was cleaved by pto-miR475a-3p/b-3p at nt 22 but by pto-miR475a-5p/b-5p at nt 110, indicative of the feedback regulatory role of miRNAs/miRNA*s on their precursors (Figure 3). pto-miR172a/b/c/f could cleave Precur-M14 at position 20 and Precur-MIR172c at position 22, falling into category 4. The downregulation of pto-miR172a/b/c/f may lead to the accumulation of Precur-M14 and Precur-MIR172c, in turn, generating more pto-M14 and pto-miR172c, thus maintaining the abundance of pto-M14 and pto-miR172c at a steady and appropriate level for further biological processes.

In addition, among these precursor-targeting miRNAs, pto-miR172a/b/c/f, pto-miR396a/b, pto-miR396c/d/e-5p, and pto-sR3 were downregulated, while pto-miR396e-3p, pto-miR475a/b-3p, and pto-sR6a accumulated during N deficiency [19], which indicates that precursor processing may play a vital role in the response of *P. tomentosa* to low N stress.

### 3.3. Identification of Biologically Functional miRNA* Strands in P. tomentosa Based on Degradome Analysis

Generally, sequences that cleave target mRNAs are annotated as mature miRNAs, and the complementary sequences within their precursors that lack cleavage function are defined as miRNA*s, which are often rapidly degraded. However, in some cases miRNA*s can accumulate to higher levels than their corresponding miRNA strands and have cleavage functions for certain targets [56]. Previous investigations showed that 28 miRNA*s in *Arabidopsis* and 18 miRNA*s in rice were more abundant than their corresponding miRNAs in certain organs (seedlings, roots, and shoot apexes), and AGO-associated silencing complex mutants [56,57,58].

The biological functions of a variety of miRNA* strands, such as miR398* and miR408* in response to *P*_i_ deficiency in *Arabidopsis* [8,59], and miR398* and miR399* in *Brassica napus* [60], have also been reported. A previous report [61] showed that miR166b*/d*, miR169i*/j*/k*, and miR396a*/b* were depressed under *P*_i_ starvation conditions in barley. These results suggested that specific miRNA*s may be involved in *P*_i_ deficiency responses instead of degradation in plants. In addition, it was reported that the repression of miR166j/k/n, miR169i/j/k, miR169i*/j*/k*, miR528a/b, and miR528a*/b* under N deficiency could play a crucial role in guiding nitrate signals involved in developmental alteration in maize roots [13]. Specifically, pto-miR169b-5p, pto-miR396e-5p, and pto-miR475a-3p/b-3p were downregulated, while pto-miR396e-3p was upregulated in response to low N stress in our previous study [20], which indicates that these miRNA*s may have an effect on the N signaling pathway in *P. tomentosa*. Interestingly, the relative abundances of mature miRNAs and miRNA* strands of specific miRNAs exhibited contrasting patterns (reduced pto-miR396e-5p and increased pto-miR396e-3p), while the high accumulation of osa-miR396a*/b* in rice roots suggested their possible role in rice root growth [57], indicative of a complex system of miRNA gene expression regulation. These results indicate that the higher abundance of miRNA*s and their biological functionality are not occasional phenomena but, more likely, are universal in plant species, and they may provide more information on miRNA or miRNA* biogenesis and regulation processes as well as provide further insights into the complex regulatory networks of miRNA systems.

## 4. Materials and Methods

### 4.1. Plant Materials

*P. tomentosa* plantlets were cultured on half-strength Murashige–Skoog (MS) medium (20 g·L^−1^ sucrose and 0.4 mg·L^−1^ indole-3-butyric acid [IBA]) under a 16/8-h (light/dark) photoperiod at 25 °C for 60 days. The plantlets were then transferred to grow in ventilated bottles for hydroponics under sufficient N conditions for 5 days, with the solution changed every 2 days. The hydroponic plantlets were then transferred to low N treatment conditions for 3 days, and those with sufficient N levels served as the control. The modified half-strength MS liquid medium for sufficient N conditions contained 2 mM NH_4_NO_3_ and 1 mM KNO_3_. The low N medium contained 0.01 mM NH_4_NO_3_and 1 mM KCl. After treatment, the plantlets were harvested intact, frozen immediately in liquid nitrogen, and stored at −80 °C until RNA extraction.

### 4.2. RNA Extraction and Degradome Sequencing

Total RNA was extracted from *P. tomentosa* plantlets using Trizol reagent (Invitrogen, Carlsbad, CA, USA). The degradome library was constructed as described previously [62]. Total RNA (200 μg) was used for degradome sequencing on an IlluminaHiSeq 2000 at the Beijing Genomics Institute (BGI), Shenzhen, China.

### 4.3. Initial Processing and Analysis of Reads for the Degradome Library

Raw data were preprocessed to remove 5′ and 3′ adaptor sequences. Low-quality reads, including tags <18 nts, those containing ambiguous bases, or those without adapter sequences, were discarded. Subsequently, the clean tags were mapped to *Populus* transcript v9.0 from the US Department of Energy Joint Genome Institute (JGI) (http://www.jgi.doe.gov/) using the SOAP software package [63]. Tags were then annotated by performing Blastn searches against the Rfam (http://www.sanger.ac.uk/Software/Rfam) and NCBI (http://www.ncbi.nlm.nih.gov/blast/Blast.cgi) data bases to ribosomal RNAs (rRNAs), transfer RNAs (tRNAs), small cytoplasmic RNAs (scRNAs), small nuclear RNAs (snRNAs), small nucleolar RNAs (snoRNAs), repeats, or polyNs, which were removed for further analysis. The remaining sequences were collected for degradome analysis. All known *Populus* miRNA mature and precursor sequences were downloaded from miRBase 20.0 (http://www.mirbase.org/ftp.shtml) or obtained from our previous investigation [19,41,42]. The blastn program option blastall in BLAST 2.2.8 was downloaded from NCBI (http://www.ncbi.nlm.nih.gov) and was used locally to map the degradome tags with *Populus* miRNA precursor sequences, using the default parameters. Perfectly matched degradome tags were considered as candidate sequences.

The identified targets were grouped into five classes based on the relative abundances of the degradome signatures at the miRNA target sites, using the CleaveLand pipeline [64], which indicates the abundance of fragments mapping at the predicted miRNA target site relative to the abundance of fragments found at other sites on the gene model. The criteria were as follows. Category 0 was defined as a cleavage site with only one maximum abundance on the transcript and with more than one raw read at the position. And targets identified in category 0 means the highest confidence targets. Category 1 was similar to category 0, but with more than one maximum position on the transcript and the confidence of targets in this category is assessed lower than that in category 0. The abundance of signatures at the cleavage site was less than the maximum but higher than the median on the transcript in category 2, which has higher confidence than the median confidence. In category 3, the abundance at the cleavage position was equal to or less than the median on the transcript. Only one raw read mapped to the splicing position in category 4 with the lowest confidence.

## 5. Conclusions

In our degradome library of *P. tomentosa* under nitrogen deficiency, 15 conserved miRNA families (23 unique miRNAs) targeted 33 mRNA transcripts, and nine non-conserved miRNA families (10 unique miRNAs) and seven novel miRNAs were confirmed to cleave 10 mRNA transcripts and seventarget genes, respectively. After removing the uncharacterized proteins, a total of 14 specific family protein genes were regulated by known and novel miRNAs, further revealing the post-transcriptional regulatory function of miRNAs. Among them, we further verified the cleavage of 11 N-responsive miRNAs identified previously, and provided empirical evidence for the cleavage mode of these miRNAs on their target mRNAs. Moreover, a total of 21 precursors were identified in our degradome. Among them, 16 precursors were targeted by seven conserved miRNA families (18 unique miRNAs), while three and two precursors were cleaved by three non-conserved miRNAs and one novel miRNA, respectively. Additionally, we identified targets of five miRNAs whose mature miRNAs and miRNA* strands both have biological cleavage functions. These results provide important information on miRNA-mediated regulation of gene expression in response to N deficiency and increase our understanding of the complex miRNA regulatory networks.

## Supplementary Materials

Supplementary materials can be found at http://www.mdpi.com/1422-0067/16/06/13937/s1.

## Data Archiving Statement

The raw sequence data can be available at the NIH Short Read Archive database (http://www.ncbi.nlm.nih.gov/sra) under the accession numberSRP056058 (Alias: PRJNA277800).
