# Supplementary material for: Transcriptome-Wide Identification of miRNA Targets under Nitrogen Deficiency in Populus tomentosa Using Degradome Sequencing
Source: Int J Mol Sci. 2015 Jun 18;16(6):13937–58. doi: 10.3390/ijms160613937 (PMC4490532; doi:10.3390/ijms160613937)
Supplement: Supplementary file 1 [file ijms-16-13937-s001.pdf]

Supplementary Information

(A) Category 0

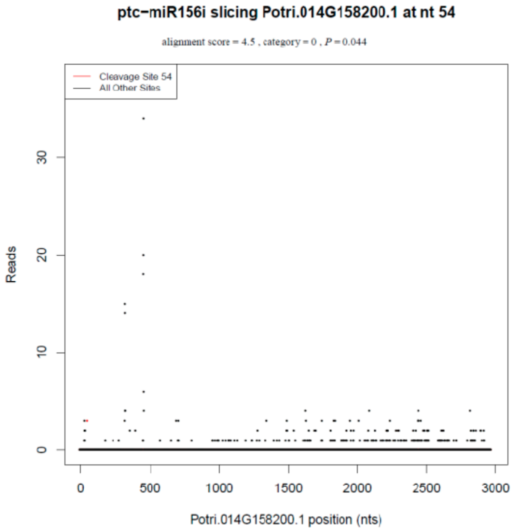

Site: 54, Score: 4.5, Category: 0

Potri.014G158200.1 5′ GCCAUCCCUCUCU−CUCUCUCUCUUCU−UUAACCAC 3′

pto-miR156i 3′ CACGAGAGAUAGAAGACAGUU 5′

(B) Category 1

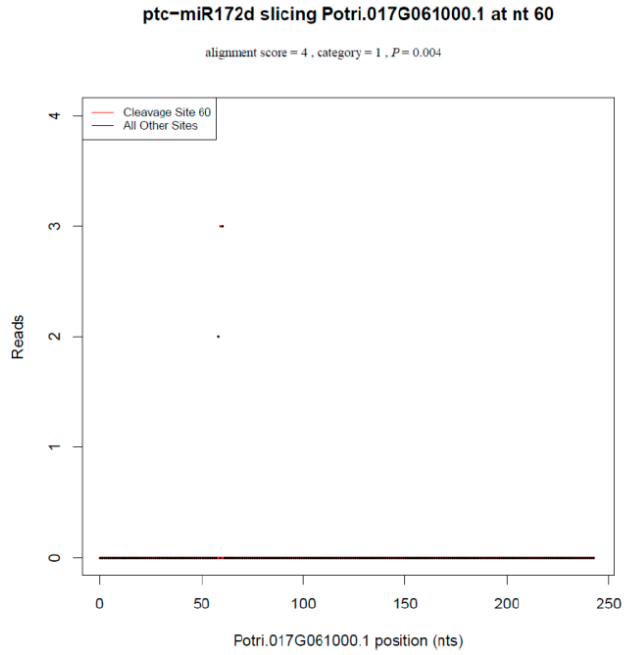

Site: 60, Score: 4, Category: 1

Potri.017G061000.1 5′ GACAGAU−CAAC−UCAUCAAG−UUCCCAUUGAA 3′

pto-miR172d 3′ UACGUCGUAGUAGUUCUAAGG 5′

Figure S1. *Cont.*

(C) Category 2

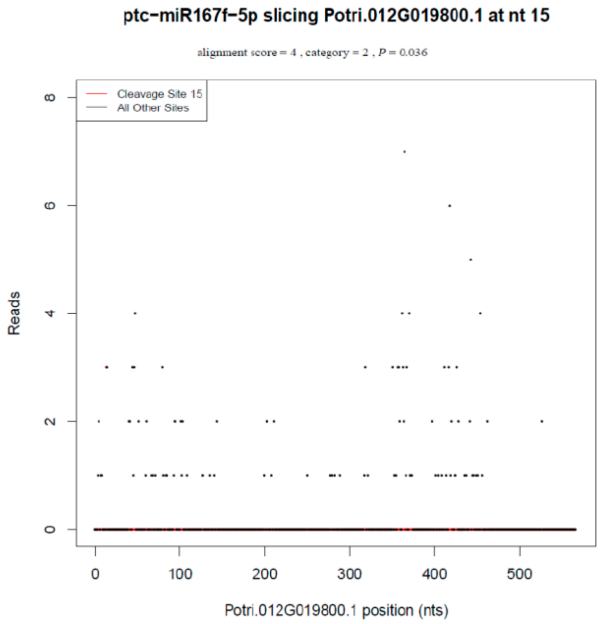

Site: 15, Score: 4, Category: 2

```
Potri.012G019800.1   5′ UUGAAAGAU−AUACUG−CAACUUCAACA 3′
                              |||||| || ||| || ||||
pto-miR167f-5p          3′ UUCUAGUACGACCGUCGAAGU 5′
```

(D) Category 3

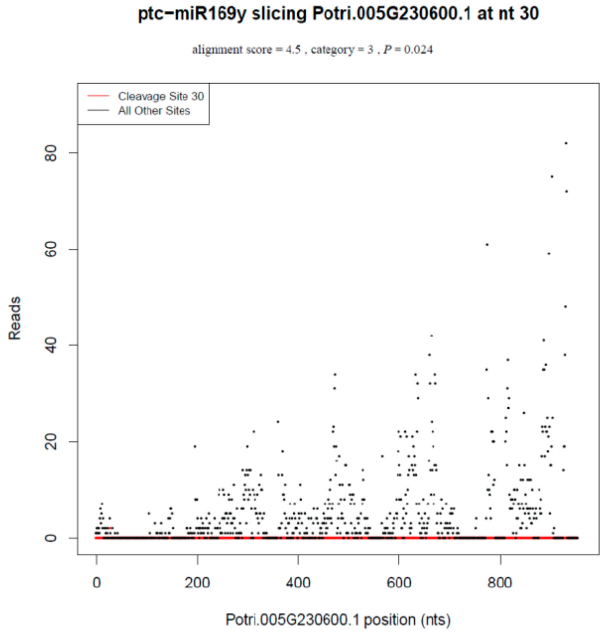

Site: 30, Score: 4.5, Category: 3

```
Potri.005G230600.1   5′ AAGGACCA−GUAAUUUAUCC−UG−CUGUGCGUGC 3
                              || |.||||.|||| || ||.
pto-miR169y              3′ GUCCGUUAAGUAGGUACCGAU 5′
```

Figure S1. *Cont.*

(E) Category 4

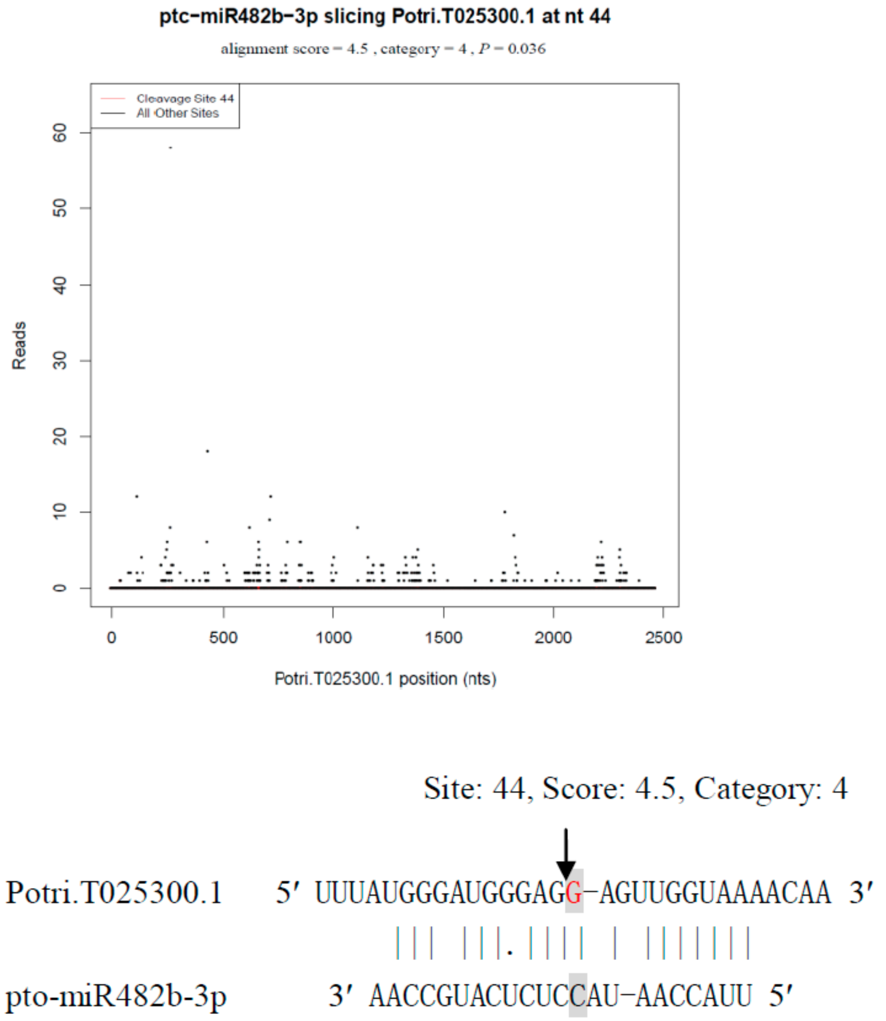

Figure S1. Target plots (*t*-plots) of miRNA targets of different categories confirmed by degradome sequencing. Representative *t*-plots for categories 0 (**A**), 1 (**B**), 2 (**C**), 3 (**D**), and 4 (**E**) are shown. Red dots indicate signatures consistent with miRNA-directed cleavage. The solid lines and dots in the miRNA:mRNA alignments indicate matched RNA base pairs and GU mismatches, respectively. Below the *t*-plots, the cleavage sites are shown by arrows and the cleaved bases identified by degradome sequencing are indicated by shaded letters.

Table S1. Total and clean read statistics of sequenced RNAs isolated from low N treated *Populus tomentosa*.

Type	Count	Percentage
Total_tags	22,047,555	100%
Clean_tags	21,906,451	99.36%
Low_quality_value	472	0.00%
Incorrect_size	59,832	0.27%
Contain_N_base	74,918	0.34%
Adaptor	5882	0.03%

Table S2. Classification of clean tags in *Populus tomentosa*.

Type	Count_of_Tags	Percentage	Unique_Count_of_Tags	Percentage
rRNA	466,426	2.13%	16,783	0.17%
tRNA	5601	0.03%	2093	0.02%
snRNA	14,285	0.07%	6665	0.07%
snoRNA	31,025	0.14%	6383	0.07%
polyN	22,805	0.10%	14,357	0.15%
cDNA_sense	11,743,449	53.61%	5,089,290	52.19%
cDNA_antisense	106,389	0.49%	29,532	0.30%
other	9,516,471	43.44%	4,586,707	47.03%
total	21,906,451	100.00%	9,751,810	100.00%

Table S3. Identification of targets and precursors for conserved, non-conserved and novel miRNAs in *Populus tomentosa*.

miRNA	Target Gene	Cleavage Site	Category	Raw Tags	Score	Target Annotation
pto-miR156g-j	Potri.014G158200.1	54	0	3	4.5	Hypothetical protein
pto-miR156g-j	Potri.014G158200.4	54	0	3	4.5	Hypothetical protein
pto-miR159a/b	Potri.006G129900.1	27	2	5	3	Peroxidase 21 precursor family protein
pto-miR160e-3p	Potri.010G060400.1	69	0	124	4.5	Hypothetical protein
pto-miR160e-3p	Potri.010G060400.2	69	0	124	4.5	Hypothetical protein
pto-miR166a-m	Potri.006G028100.1	44	4	1	4.5	Hypothetical protein
pto-miR166n/o/q	Potri.007G119200.2	37	2	2	4	Hypothetical protein
pto-miR166n/o/q	Potri.006G028100.1	44	4	1	4	Hypothetical protein
pto-miR167f/g-5p	Potri.012G019800.1	15	2	3	4	Hypothetical protein
pto-miR169a/b-5p/c	Potri.007G127100.1	31	1	2	4.5	Hypothetical protein
pto-miR169b-3p	Potri.007G085200.1	60	2	8	3.5	60S ribosomal protein L26B
pto-miR169b-3p	Potri.010G155300.1	45	2	2	4.5	Hypothetical protein
pto-miR169b-3p	Potri.014G142600.1	38	2	2	4	ORESARA 9 family protein
pto-miR169v/w	Potri.007G127100.1	31	1	2	4.5	Hypothetical protein
pto-miR169y	Potri.005G230600.1	30	3	2	4.5	Wound-responsive family protein
pto-miR172d/e	Potri.017G061000.1	60	1	3	4	Hypothetical protein
pto-miR395a	Potri.002G092400.1	56	0	11	4.5	Low affinity sulfate transporter 3 family protein
pto-miR395a	Potri.002G092400.2	56	0	11	4.5	Low affinity sulfate transporter 3 family protein
pto-miR395a	Potri.002G092400.4	56	0	11	4.5	Low affinity sulfate transporter 3 family protein
pto-miR395a	Potri.002G092400.5	56	0	11	4.5	Low affinity sulfate transporter 3 family protein
pto-miR395a	Potri.002G092400.6	56	0	11	4.5	Low affinity sulfate transporter 3 family protein
pto-miR395a	Potri.002G092400.7	56	0	11	4.5	Low affinity sulfate transporter 3 family protein
pto-miR395b-k	Potri.002G092400.1	56	0	11	2	Low affinity sulfate transporter 3 family protein
pto-miR395b-k	Potri.002G092400.2	56	0	11	2	Low affinity sulfate transporter 3 family protein
pto-miR395b-k	Potri.002G092400.4	56	0	11	2	Low affinity sulfate transporter 3 family protein
pto-miR395b-k	Potri.002G092400.5	56	0	11	2	Low affinity sulfate transporter 3 family protein
pto-miR395b-k	Potri.002G092400.6	56	0	11	2	Low affinity sulfate transporter 3 family protein
pto-miR395b-k	Potri.002G092400.7	56	0	11	2	Low affinity sulfate transporter 3 family protein
pto-miR395b-k	Potri.002G092400.3	56	2	11	2	Low affinity sulfate transporter 3 family protein
pto-miR396g-5p	Potri.017G062800.2	36	2	2	4	Shikimate kinase family protein
pto-miR396h	Potri.018G026100.1	50	1	6	4.5	Hypothetical protein

Table S3. *Cont.*

miRNA	Target Gene	Cleavage Site	Category	Raw Tags	Score	Target Annotation
pto-miR472a	Potri.T028700.1	46	0	111	3	Hypothetical protein
pto-miR472a	Potri.T025300.1	46	4	1	3	Hypothetical protein
pto-miR472b	Potri.T028700.1	46	0	111	2	Hypothetical protein
pto-miR472b	Potri.T025300.1	46	4	1	2	Hypothetical protein
pto-miR472b	Potri.T025500.1	25	4	1	3	Hypothetical protein
pto-miR472b	Potri.T026600.1	46	4	1	3	Hypothetical protein
pto-miR476b	Potri.001G028800.1	59	1	2	4	Hypothetical protein
pto-miR477a-3p	Potri.001G289500.1	43	3	2	3.5	TUBULIN ALPHA CHAIN family protein
pto-miR478f	Potri.003G091700.1	42	4	1	4.5	Hypothetical protein
pto-miR482a.2	Potri.T028700.1	46	0	111	3.5	Hypothetical protein
pto-miR482a.2	Potri.019G014500.1	10	2	2	3.5	Hypothetical protein
pto-miR482a.2	Potri.T028300.1	10	2	2	4	Hypothetical protein
pto-miR482a.2	Potri.T025300.1	46	4	1	3	Hypothetical protein
pto-miR482a.2	Potri.T025500.1	25	4	1	3.5	Hypothetical protein
pto-miR482a.2	Potri.T026600.1	46	4	1	3.5	Hypothetical protein
pto-miR482b-3p	Potri.T025300.1	44	4	1	4.5	Hypothetical protein
pto-miR1446a-e	Potri.005G129400.1	10	1	3	4.5	Hypothetical protein
pto-miR1449	Potri.005G050500.1	18	2	26	4	Helicase domain-containing family protein
pto-miR6427-3p	Potri.001G014000.1	30	2	2	3.5	Hypothetical protein
pto-miR6427-3p	Potri.001G014000.2	28	2	2	3.5	LIPOYLTRANSFERASE 2 family protein
pto-miR6427-3p	Potri.001G014000.3	30	2	2	3.5	LIPOYLTRANSFERASE 2 family protein
pto-miR6427-3p	Potri.010G071900.1	28	2	3	4.5	Hypothetical protein
pto-miR6439a	Potri.013G063100.1	50	2	2	3	Germin-like protein 1
pto-miR6439a	Potri.013G063200.1	50	2	2	3	Germin-like protein 1
pto-miR6439a	Potri.013G063300.1	50	2	2	3	Germin-like protein 1
pto-miR6439a	Potri.013G063500.1	50	2	2	3	Germin-like protein 1
pto-miR6439a	Potri.013G063600.1	50	2	2	3	Germin-like protein 1
pto-miR6439a	Potri.013G063700.1	50	2	2	3	Germin-like protein 1
pto-miR6439a	Potri.013G063800.1	50	2	2	3	Germin-like protein 1
pto-miR6439a	Potri.013G064100.1	50	2	2	3	Hypothetical protein
pto-miR6439b	Potri.001G161700.1	29	4	1	4	26S protease regulatory subunit 7 family protein
pto-miR6439b	Potri.001G161700.2	40	4	1	4	26S protease regulatory subunit 7 family protein
pto-miR6439b	Potri.001G161700.3	29	4	1	4	26S protease regulatory subunit 7 family protein
pto-miR6439b	Potri.001G161700.4	29	4	1	4	26S protease regulatory subunit 7 family protein
pto-miR6462c-5p	Potri.012G018600.2	13	2	3	3.5	hypothetical protein
pto-miR6463	Potri.009G134200.4	57	2	3	3.5	C2 domain-containing family protein
pto-miR6470	Potri.010G228700.1	51	2	3	3	Glycine-rich RNA-binding family protein
pto-miR6479	Potri.001G452600.1	64	1	2	4.5	Oxidoreductase family protein
pto-miR7828	Potri.015G101200.1	64	2	2	4	Hypothetical protein
pto-miR7841	Potri.010G048600.1	29	0	3	4.5	Hypothetical protein
pto-miR7841	Potri.010G048600.2	29	0	3	4.5	Hypothetical protein
pto-miRS11	Potri.010G066400.3	71	2	10	2.5	Hypothetical protein
pto-miRS11	Potri.010G066400.3	68	2	3	2.5	Hypothetical protein
pto-miRS11	Potri.010G066400.3	69	3	2	2.5	Hypothetical protein

Table S3. *Cont.*

miRNA	Target Gene	Cleavage Site	Category	Raw Tags	Score	Target Annotation
pto-miRS11	Potri.010G066400.3	70	3	2	2.5	Hypothetical protein
pto-miRS11	Potri.010G066400.3	67	4	1	2.5	Hypothetical protein
pto-miRS11	Potri.010G066400.3	66	4	1	2.5	Hypothetical protein
pto-miRS11	Potri.010G066400.3	72	4	1	3.5	Hypothetical protein
pto-sM3	Potri.012G089700.1	31	4	1	4	Hypothetical protein
pto-smR5	Potri.018G009400.5	54	4	1	3.5	Hypothetical protein
pto-sR2	Potri.010G074100.1	45	1	3	4.5	Hypothetical protein
pto-SR22	Potri.012G041600.1	11	4	1	3.5	Hypothetical protein
pto-M19	Potri.007G090600.1	69	3	2	4	Unknown
pto-M53a	Potri.011G065200.1	25	2	4	3	Hypothetical protein
pto-miR168a/b-3p	Precur-MIR168a	20	4	1	4	Hypothetical protein
pto-miR168a/b-3p	Precur-MIR168b	20	4	1	4	Hypothetical protein
pto-miR172a-c/f	Precur-M14	20	4	1	1.5	–
pto-miR172a-c/f	Precur-MIR172c	22	4	1	1.5	–
pto-miR172d/e	Precur-M14	20	4	1	1.5	–
pto-miR172d/e	Precur-MIR172c	22	4	1	1.5	–
pto-miR172i	Precur-M14	20	4	1	3	–
pto-miR172i	Precur-MIR172c	22	4	1	3	–
pto-miR319a-d	Precur-MIR159a	20	3	2	4	–
pto-miR319e-h	Precur-MIR159a	20	3	2	4	–
pto-miR319i	Precur-MIR159a	20	3	2	4.5	–
pto-miR396a/b	Precur-sR3	118	0	28	2	–
pto-miR396c/d/e-5p	Precur-sR6a	127	2	7	3.5	–
pto-miR396e-3p	Precur-MIR396a	19	2	5	1.5	–
pto-miR396e-3p	Precur-MIR396b	19	2	5	1.5	–
pto-miR396e-3p	Precur-MIR396e	19	4	1	2	–
pto-miR396e-3p	Precur-sR3	22	2	5	1.5	–
pto-miR396e-3p	Precur-sR6a	19	4	1	2	–
pto-miR396f	Precur-sR3	118	0	28	2.5	–
pto-miR396g-5p	Precur-sR6a	127	2	7	4	–
pto-miR398b/c-3p	Precur-MIR398c	22	0	49	3	–
pto-miR398c-5p	Precur-MIR398b	65	1	2	3	–
pto-miR398c-5p	Precur-MIR398c	77	2	2	3	–
pto-miR408-5p	Precur-MIR408	85	2	2	2.5	Hypothetical protein
pto-miR475a/b/d-5p	Precur-MIR475b	110	2	3	3	–
pto-miR475a/b-3p	Precur-MIR475a	15	4	1	2	–
pto-miR475a/b-3p	Precur-MIR475b	22	4	1	2	–
pto-miR475a/b-3p	Precur-MIR475d	19	4	1	3	–
pto-miR475c	Precur-MIR475a	15	4	1	1.5	–
pto-miR475c	Precur-MIR475b	22	4	1	1.5	–
pto-miR475c	Precur-MIR475d	19	4	1	2.5	–
pto-miR1447	Precur-MIR1447	20	4	1	1.5	Hypothetical protein
pto-miR1450	Precur-MIR1450	34	2	15	2	–
pto-miR6421-5p	Precur-MIR6421	93	4	1	2	–
pto-miRS12	Precur-MIR171e	71	0	2	3.5	–
pto-miRS12	Precur-sR7a	71	0	2	3.5	–

Table S4. Targets cleavage validation of identified N-responsive miRNAs (Ren *et al.* 2015) in *Populus tomentosa*.

MiRNA	Regulation	MiRNA Sequence	Target Gene	C-Sites	Category	Rawtags	Score	Target Annotation
pto-miR159a/b	Down	TTTGGATTGAAGGGAGCTCTA	Potri.006G129900.1	27	2	5	3	Peroxidase 21 precursorfamily protein
pto-miR160e-3p	Down	GCATGAGGGGAGTCGAGCAGG	Potri.010G060400.1	69	0	124	4.5	Hypothetical protein
pto-miR160e-3p	Down	GCATGAGGGGAGTCGAGCAGG	Potri.010G060400.2	69	0	124	4.5	Hypothetical protein
pto-miR166a-m	Down	TCGGACCAGGCTTCATTCCCC	Potri.006G028100.1	44	4	1	4.5	Hypothetical protein
pto-miR169a/b-5p/c	Down	CAGCCAAGGATGACTTGCCGA	Potri.007G127100.1	31	1	2	4.5	Hypothetical protein
pto-miR172a/b-3p/c/f	Down	AGAATCTTGATGATGCTGCAT	Precur-M14	20	4	1	1.5	–
pto-miR172a/b-3p/c/f	Down	AGAATCTTGATGATGCTGCAT	Precur-MIR172c	22	4	1	1.5	–
pto-miR395b-k	Up	CTGAAGTGTTTGGGGGAACTC	Potri.002G092400.1	56	0	11	2	Low affinity sulfate transporter 3 family protein
pto-miR395b-k	Up	CTGAAGTGTTTGGGGGAACTC	Potri.002G092400.2	56	0	11	2	Low affinity sulfate transporter 3 family protein
pto-miR395b-k	Up	CTGAAGTGTTTGGGGGAACTC	Potri.002G092400.3	56	2	11	2	Low affinity sulfate transporter 3 family protein
pto-miR395b-k	Up	CTGAAGTGTTTGGGGGAACTC	Potri.002G092400.4	56	0	11	2	Low affinity sulfate transporter 3 family protein
pto-miR395b-k	Up	CTGAAGTGTTTGGGGGAACTC	Potri.002G092400.5	56	0	11	2	Low affinity sulfate transporter 3 family protein
pto-miR395b-k	Up	CTGAAGTGTTTGGGGGAACTC	Potri.002G092400.6	56	0	11	2	Low affinity sulfate transporter 3 family protein
pto-miR395b-k	Up	CTGAAGTGTTTGGGGGAACTC	Potri.002G092400.7	56	0	11	2	Low affinity sulfate transporter 3 family protein
pto-miR396a/b	Up	TTCCACAGCTTTCTTGAACTG	Precur-sR3	118	0	28	2	–
pto-miR396c/d/e-5p	Down	TTCCACAGCTTTCTTGAACTT	Precur-sR6a	127	2	7	3.5	–
pto-miR396e-3p	Up	CTCAAGAAAGCTGTGGGAGA	Precur-MIR396a	19	2	5	1.5	–
pto-miR396e-3p	Up	CTCAAGAAAGCTGTGGGAGA	Precur-MIR396b	19	2	5	1.5	–
pto-miR396e-3p	Up	CTCAAGAAAGCTGTGGGAGA	Precur-MIR396e	19	4	1	2	–
pto-miR396e-3p	Up	CTCAAGAAAGCTGTGGGAGA	Precur-sR3	22	2	5	1.5	–
pto-miR396e-3p	Up	CTCAAGAAAGCTGTGGGAGA	Precur-sR6a	19	4	1	2	–
pto-miR475a-3p/b-3p	Down	TTACAGTGCCCATTGATTAAG	Precur-MIR475a	15	4	1	2	–
pto-miR475a-3p/b-3p	Down	TTACAGTGCCCATTGATTAAG	Precur-MIR475b	22	4	1	2	–
pto-miR475a-3p/b-3p	Down	TTACAGTGCCCATTGATTAAG	Precur-MIR475d	19	4	1	3	–
pto-miR6427-3p	Down	GTGGGAATGAACATTATGAGA	Potri.001G014000.1	30	2	2	3.5	Hypothetical protein
pto-miR6427-3p	Down	GTGGGAATGAACATTATGAGA	Potri.001G014000.2	28	2	2	3.5	LIPOYLTRANSFERASE 2 family protein
pto-miR6427-3p	Down	GTGGGAATGAACATTATGAGA	Potri.001G014000.3	30	2	2	3.5	LIPOYLTRANSFERASE 2 family protein
pto-miR6427-3p	Down	GTGGGAATGAACATTATGAGA	Potri.010G071900.1	28	2	3	4.5	Hypothetical protein

Table S5. Identification of the different cleavage sites for the same mRNA target.

miRNA	Target Gene	C-Site	Category	Rawtags	Score
pto-miRS11	Potri.010G066400.3	66	4	1	2.5
pto-miRS11	Potri.010G066400.3	67	4	1	2.5
pto-miRS11	Potri.010G066400.3	68	2	3	2.5
pto-miRS11	Potri.010G066400.3	69	3	2	2.5
pto-miRS11	Potri.010G066400.3	70	3	2	2.5
pto-miRS11	Potri.010G066400.3	71	2	10	2.5
pto-miRS11	Potri.010G066400.3	72	4	1	3.5
pto-miR472a	Potri.T025300.1	46	4	1	3
pto-miR472b	Potri.T025300.1	46	4	1	2
pto-miR482a.2	Potri.T025300.1	46	4	1	3
pto-miR482b-3p	Potri.T025300.1	44	4	1	4.5
pto-miR398b/c-3p	Precursor-MIR398c	22	0	49	3
pto-miR398c-5p	Precursor-MIR398c	77	2	2	3
pto-miR396e-3p	Precursor-sR3	22	2	5	1.5
pto-miR396a/b	Precursor-sR3	118	0	28	2
pto-miR396f	Precursor-sR3	118	0	28	2.5
pto-miR396e-3p	Precursor-sR6a	19	4	1	2
pto-miR396c/d/e-5p	Precursor-sR6a	127	2	7	3.5
pto-miR396g-5p	Precursor-sR6a	127	2	7	4
pto-miR475a-3p/b-3p	Precursor-MIR475b	22	4	1	2
pto-miR475c	Precursor-MIR475b	22	4	1	1.5
pto-miR475a-5p/b-5p/d-5p	Precursor-MIR475b	110	2	3	3

C-Site represents “cleavage site.
